# Clinical Significance of Nuclear Yin-Yang Overexpression Evaluated by Immunohistochemistry in Tissue Microarrays and Digital Pathology Analysis: A Useful Prognostic Tool for Breast Cancer

**DOI:** 10.3390/ijms26188777

**Published:** 2025-09-09

**Authors:** Mayra Montecillo-Aguado, Giovanny Soca-Chafre, Gabriela Antonio-Andres, Belen Tirado-Rodriguez, Daniel Hernández-Cueto, Clara M. Rivera-Pazos, Marco A. Duran-Padilla, Sandra G. Sánchez-Ceja, Berenice Alcala-Mota-Velazco, Anel Gomez-Garcia, Sergio Gutierrez-Castellanos, Sara Huerta-Yepez

**Affiliations:** 1 Unidad de Investigacion en Enfermedades Oncologicas, Hospital Infantil de Mexico, Federico Gomez, Mexico City 06720, Mexico; mayramontecillo@gmail.com (M.M.-A.); giovsoch@gmail.com (G.S.-C.); gabya_24@yahoo.com.mx (G.A.-A.); abtirado81@gmail.com (B.T.-R.); danteligueri@gmail.com (D.H.-C.); monsserp@yahoo.com.mx (C.M.R.-P.); 2 Laboratorio de Biomedicina Traslacional, División Ciencias de la Salud, Universidad de Guanajuato, Leon Guanajuato 36000, Mexico; 3 Unidad de Patología Quirúrgica, Hospital General de México Dr. Eduardo Liceaga, Mexico City 04510, Mexico; patologiaduran@hotmail.com; 4 Laboratorio de Patología, Facultad de Químico Farmacobiología, Universidad Michoacana de San Nicolás de Hidalgo (UMSNH), Morelia 58060, Mexico; sgsanchezc@gmail.com (S.G.S.-C.); sergio.gutierrezc@imss.gob.mx (S.G.-C.); 5 Departamento de Patología, Facultad de Odontología, Universidad Michoacana de San Nicolás de Hidalgo (UMSNH), Morelia 58060, Mexico; bamota@umich.mx; 6 Centro de investigación Biomédica de Michoacán, División de Investigación Clínica, Instituto Mexicano del Seguro Social, Morelia 58060, Mexico; anel.gomez@imss.gob.mx

**Keywords:** breast cancer, YY1 protein expression, overall survival, digital pathology, tissue microarray

## Abstract

Yin Yang 1 (YY1) is a multifunctional transcription factor implicated in gene regulation, cell proliferation, and survival. While its role in breast cancer (BC) has been explored, its prognostic significance remains controversial. In this study, we evaluated nuclear YY1 expression in 276 BC tissue samples using immunohistochemistry (IHC), tissue microarrays (TMAs), and digital pathology (DP). Nuclear staining was quantified using Aperio ImageScope software, focusing on tumor regions to avoid confounding from stromal or non-tumor tissues. This selective and standardized approach enabled precise quantification of YY1 expression. Our results show elevated median YY1 expression in tumor vs. normal matched tissues (*p* < 0.001). The optimal cutoff for medium-intensity nuclear YY1 expression in tumor areas for overall survival (OS) was established by a receiver operating characteristic (ROC) curve (AUC = 0.718, 95% CI: 0.587–0.849, *p* = 0.008). In contrast, ROC curves showed no prognostic impact (AUC and *p*-value) for YY1 quantification in whole spots (tumor + normal). As a categorical variable, high YY1 expression was correlated with more aggressive BC features, including tumor size > 3 cm (57.7% vs. 44.2% *p* = 0.037), the triple-negative breast cancer (TNBC) molecular subtype (27.3% vs. 13.9% *p* = 0.026), and advanced prognostic stage (III) (31.8% vs. 16.7% *p* = 0.003), while as a continuous variable, YY1 was associated with higher histological (*p* = 0.003) and nuclear grades (*p* = 0.022). High YY1 expression was significantly associated with a reduced OS of BC patients, as shown by Kaplan–Meier curves (HR = 2.227, *p* = 0.002). Since YY1 was significantly enriched in TNBC, we evaluated its prognostic resolution in this subgroup. But, probably due to the small number of patients within this subset, our results were not statistically significant (HR = 1.317, 95% CI: 0.510–3.405, *p* = 0.566). Next, we performed multivariate Cox regression, confirming YY1 as an independent prognostic factor for overall survival (HR = 1.927, 95% CI: 1.144–3.247, *p* = 0.014). In order to improve prognostic value, we constructed a mathematical model derived from the multivariate Cox regression results, including YYI, AJCC prognostic stage (STA), and axillary lymph node dissection (ALN), with the following equation: h(t) = h_0_(t) × exp (0.695 × YY1 + 1.103 × STA − 0.503 × ALN). ROC analysis of this model showed a better AUC of 0.915, similar sensitivity (83.3%), and much higher specificity (92%). Bioinformatic analysis of public datasets supported these findings in BC, showing YY1 overexpression in multiple cancer types and its association with poor outcomes in BC. These results suggest that YY1 may play a role in tumor progression and serve as a valuable prognostic biomarker in BC. DP combined with molecular data enhanced biomarker accuracy, supporting clinical applications of YY1 in routine diagnostics and personalized therapy. Additionally, developing a combined score based on the modeling of multiple prognostic factors significantly enhanced survival predictions, representing a practical tool for risk stratification and the guidance of therapeutic decisions.

## 1. Introduction

Breast cancer (BC) remains one of the most prevalent and life-threatening malignancies among women worldwide. In 2020 alone, it accounted for 11.7% of all cancer cases globally, with an estimated 2.3 million new diagnoses [1]. Alarmingly, projections suggest that this number will surpass 3 million cases annually by 2040 [2]. Among its subtypes, triple-negative breast cancer (TNBC) is particularly aggressive and lacks targeted therapies, leading to poor prognosis. More than 50% of early-stage TNBC patients relapse, and 37% die within five years of diagnosis [3]. In metastatic TNBC, mortality reaches approximately 50% within the first year after diagnosis [4,5].

BC development is driven by the deregulation of key tumor suppressors and oncogenes, which disrupt crucial molecular pathways [6,7,8]. Despite advances in the understanding of gene function, targeted therapies remain limited due to challenges such as insufficient tumor specificity and limited durability of response, highlighting the need for novel molecular targets.

Yin Yang 1 (YY1) is a multifunctional transcription factor regulating approximately 10% of mammalian genes [9]. It is involved in cell proliferation, survival, apoptosis, and migration [10,11]. YY1 overexpression has been reported in various malignancies, including both pediatric [12,13,14] and adult cancers, such as hepatocellular carcinoma, prostate cancer, bladder cancer, and BC [15,16,17,18]. Specifically, in BC, YY1 expression is elevated and associated with disease progression [19,20]. Clinically, high YY1 levels have been related to poor outcomes, shorter overall survival (OS), and an increased risk of metastasis [12,13,14].

According to GLOBOCAN (https://gco.iarc.fr, accessed on 8 September 2025), BC has the second highest global incidence and the fourth-highest mortality rate among all cancers. BC is classified into three primary subtypes: hormone receptor-positive BC, HER2-positive BC, and TNBC. Each subtype presents unique histopathological features and clinical outcomes [21]. Interestingly, YY1 is overexpressed in all major BC subtypes compared to normal tissue, with the highest expression observed in luminal B tumors [22].

Functional studies in vitro support YY1’s oncogenic role. Its depletion reduces the migration, invasion, and tumorigenic potential of breast cancer cell lines [23]. YY1 enhances epithelial–mesenchymal transition (EMT) by regulating kinectin expression and promotes tamoxifen resistance via the circ_0001946/miR-671-5p/EGFR axis [24]. Moreover, YY1 suppresses apoptosis in TNBC by downregulating pro-apoptotic genes like JAW [25] and activating lncRNA DUXAP8 [26]. SETD7-mediated stabilization of YY1 further promotes EMT in TNBC [23]. High levels of circ_0001946 and SETD7 in patient biopsies correlate with worse outcomes [23,27].

Despite compelling evidence of YY1 promoting aggressive cancer behavior, its prognostic significance remains controversial. Many studies have linked YY1 overexpression to poor clinical outcomes [26,28]. For example, YY1 overexpression has been associated with adverse prognosis through the transcriptional regulation of several oncogenic molecules, including the long intergenic non-protein-coding RNA 673 (LINC00673) [29], the long non-coding RNA double homeobox A pseudogene 8 (DUXAP8), the family with sequence similarity 111 member B (FAM111B) oncoprotein [30], and the defective in cullin neddylation 1 domain containing 5 (DCUN1D5) protein in TNBC [31].

In contrast, other studies have suggested an opposite role for YY1 in breast cancer prognosis. Notably, Cha et al. (2023) [32] reported that nuclear YY1 overexpression was significantly correlated with favorable clinicopathological features and improved survival in luminal (ER+/HER2−) breast cancer patients. In their cohort of 491 patients, high YY1 expression was associated with better 5-year overall and disease-free survival, and multivariate analysis confirmed YY1 as an independent marker of good prognosis. Similarly, Cha et al. demonstrated that, after a median follow-up of 68 months, YY1 overexpression was associated with improved outcomes. Using a Cox proportional hazards model, they further showed that YY1 overexpression was an independent prognostic factor by adjusting for hormone receptor/HER2 status and tumor size [33].

Given these discrepancies, it is crucial to clarify YY1’s prognostic value. In this study, we analyzed nuclear YY1 expression in a cohort of 276 Mexican women with breast cancer through tissue microarrays (TMAs), immunohistochemistry (IHC), and digital pathology (DP). Our goal was to evaluate YY1’s prognostic role with greater precision by quantifying its expression exclusively in tumor regions, minimizing bias from surrounding stromal tissues.

## 2. Results

### 2.1. Clinical Profile and Pathological Features of the Study Population

In this study, we retrospectively analyzed clinical data from 276 patients diagnosed with primary invasive breast cancer (BC) between 2015 and 2017 (Table 1). Some variables displayed a slightly different number of cases due to missing data. Age ranged from 25 to 86 years, with a median of 52 and nearly 60% at or above 50 years. Most cases exhibited higher histological (II + III: 78.4%) and nuclear grades (2 + 3: 90.8%), characteristic of less differentiated and more aggressive tumors. Non-infiltrative borders were present in three-quarters of the cohort, while half of the cohort presented tumor diameter ≥ 3 cm, often referred to as T2 or T3. Axillary lymph nodes were dissected in over 60% of patients. Most patients were estrogen receptor-positive (ER+), progesterone receptor-positive (PR+), and HER2-negative, representing around 70, 60, and 80%, respectively. Luminal (A + B) molecular subtypes were predominant (~70%), followed by TNBC (~20%). In this cohort, patients were mostly diagnosed at early stages (I and II), accounting for 75% of cases, while four out of five patients (~80%) did not experience recurrence.

Patients were then stratified into high and low YY1 protein expression (Table 2) based on a biomarker cutoff value of 5.5743860 × 10^7^ pixels, as described in the section below. Multiple clinical and pathological variables were dichotomized to explore significant associations with YY1 expression. Among this cohort, 111 patients (40.2%) exhibited high and 165 (59.7%) exhibited low YY1 expression. Most clinical characteristics were not related to YY1 expression; however, we found that a larger tumor size, a feature with an elevated risk of spread, was significantly associated with a high YY1 value (57.7 vs. 44.2%, *p* = 0.037). Conversely, ALND showed a borderline association with low-YY1 patients (68.5 vs. 57.7%, *p* < 0.1). Interestingly, although luminal A + B was the most frequent molecular subtype, the high-YY1 subset was significantly enriched in TNBC, with almost twice the frequency than in the low-YY1 subgroup (27.3 vs. 13.9%, *p* = 0.026). Likewise, although early prognostic stages were the most abundant in this cohort, advanced stage III was significantly associated with high-YY1 patients (31.8 vs. 16.7%), whereas early stage I was predominant in low-YY1 patients (45.7 vs. 26.4%, *p* = 0.003).

Besides these categorical associations, YY1 expression as a quantitative variable was elevated in advanced histological (*p* = 0.005) and nuclear grades (*p* = 0.022) while less expressed (borderline trend) in ER+ patients (*p* = 0.097) and tumors with infiltrative borders (*p* = 0.085). YY1 also exhibited a borderline correlation with patients in the overweight range (body mass index ≥ 28.4) (*p* = 0.052) (Supplementary Figure S1).

### 2.2. Digital Pathology Approach for Quantification of YY1 and Cutoff Point

**Figure 1:**
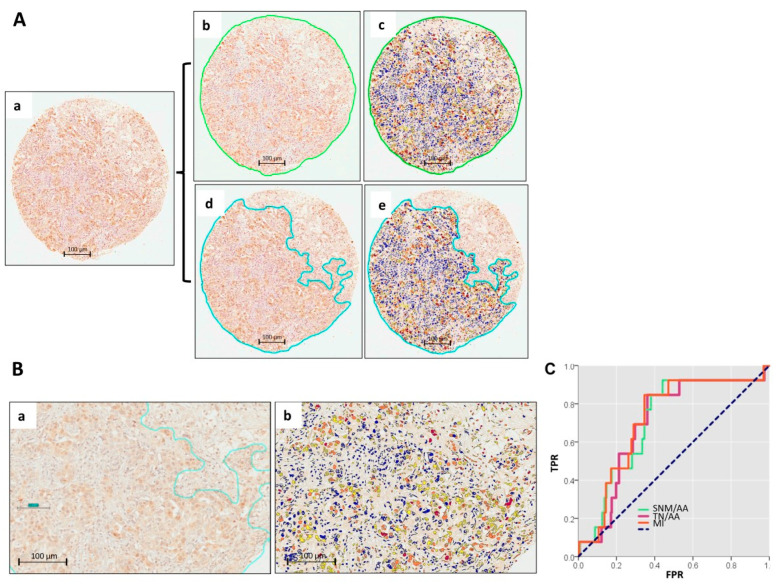
Analysis of YY1 expression by digital pathology (DP) in TMAs from breast cancer (BC) patients. (**A**) Representative TMA spot from a BC patient (**a**); in this spot, we analyzed YY1 expression using two different approaches: (**b**) The whole spot was selected (outlined in green), including tumor, normal, and inflammatory infiltrates, and YY1 expression was quantified using DP (**c**). In (**d**), only the tumor area (outlined in blue) was selected, and YY1 expression was evaluated exclusively within this region by DP (**e**). Scale 100 μm. (**B**) Representative microphotograph of YY1 staining at higher magnification (**a**), showing both nuclear and cytoplasmic localization. (**b**) Differential staining through IHC analyzed by DP: red = high, orange = medium, yellow = low, and blue = negative expression. Scale 100 µm. (**C**) ROC curve analysis of selected variables for YY1 quantification by DP.

We measured YY1 protein expression in a cohort of 276 BC patients based on IHC of TMAs. We found a clear nuclear localization of YY1 in tumor cells by IHC with occasional cytoplasmic staining. Interestingly, nuclear YY1 expression was notably intense in malignant cells (Figure 1A(b)), and we also detected YY1-positive nuclei in infiltrating immune cells within the tumor microenvironment (Figure 1A(c)). In previous studies by our research group, we demonstrated how an analysis of the tumor area—rather than of the whole (tumor + normal) area—yields more robust and clinically relevant results [34]. Based on these findings, we applied a targeted DP approach in this study. Using the Aperio ImageScope system with the guidance of two expert pathologists, we precisely delineated tumor areas within each TMA spot for digital quantification (Figure 1A(d,e)). This selective analysis allowed for a more consistent and accurate assessment of nuclear YY1 expression, minimizing the variability introduced by surrounding normal tissue.

Figure 1B illustrates representative IHC staining of YY1 in BC tissue. As shown in Figure 1B(a), YY1 displays differential nuclear expression within tumor areas (outlined in green). Using Aperio ImageScope software, we were able to accurately distinguish between low, medium, and high levels of nuclear YY1 expression (Figure 1B(b)).

Based on this stratified expression, we screened candidate variables as potential prognostic biomarkers. Testing all variables for YY1 quantification by receiver operating characteristic (ROC) curves, we found potential prognostic factors among those within the tumor areas but not in the whole spots (tumor + normal) (Supplementary Figure S2A and Figure S2B, respectively). Three YY1-related quantitative variables were derived from tumor areas and analyzed: medium intensity (MI), strong nuclei plus mean/average area (SNM/AA), and total nuclei per average area (TN/AA) (Figure 1C). Among these, medium intensity (MI) emerged as the strongest candidate, displaying better prognostic value, i.e., a higher area under the curve value (AUC = 0.718) and statistical significance (*p* = 0.008). The optimal cutoff point for MI was set to 5.5743860 × 10^7^ pixels, maximizing sensitivity (85%) and specificity (67%).

**Figure 2:**
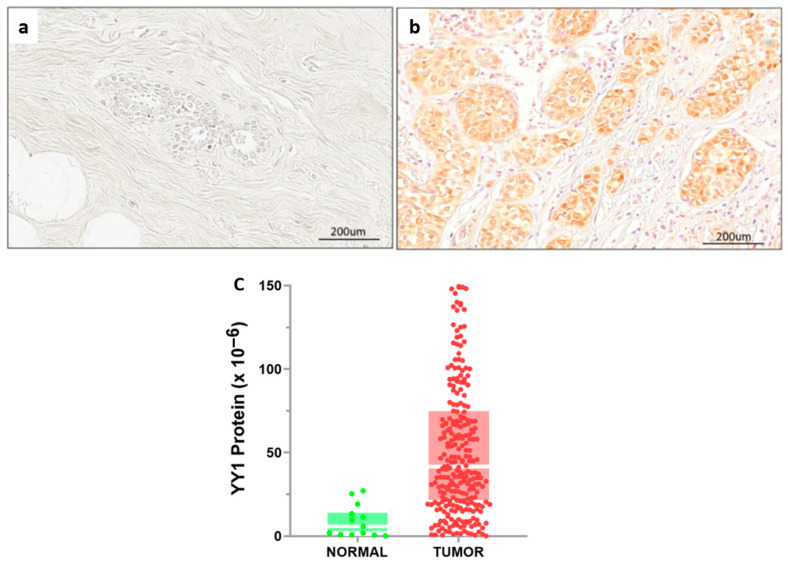
YY1 protein expression and DP analysis of TMAs from BC patients: Representative IHC-stained slides for normal (**a**) vs. tumor tissue (**b**). (**c**) Quantitative statistical analysis by Mann–Whitney U test reveals significantly higher YY1 expression in matched tumor vs. normal tissue (*p* < 0.001).

Then, we explored the clinical relevance of YY1 in BC, comparing nuclear protein expression in matched tumor and normal BC samples from our cohort. IHC analysis revealed markedly lower YY1 nuclear staining in normal breast epithelial cells (Figure 2a) than in tumor cells, where strong nuclear expression was consistently observed (Figure 2b). Quantitative analysis of YY1 expression confirmed significant upregulation in tumor vs. normal tissues (Figure 2c). Altogether, these results support the hypothesis that YY1 is overexpressed in malignant breast tissue and plays a potential role in tumorigenesis and tumor progression.

### 2.3. Prognostic Value of Nuclear YY1 Expression: Univariate and Multivariate Survival Analyses

**Figure 3:**
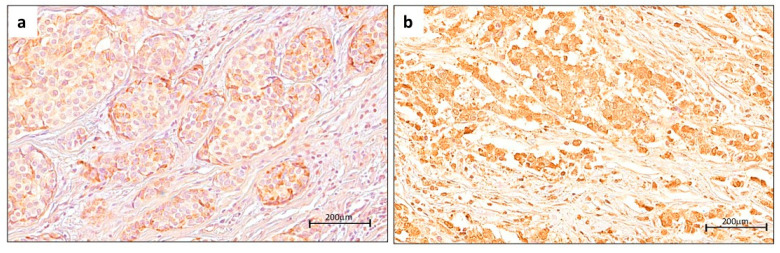
Nuclear protein expression levels of YY1 measured by IHC staining and DP in tumor samples from TMAs of BC patients. (**a**) Low YY1 expression. (**b**) High YY1 expression (scale bar = 200 μm).

To evaluate the prognostic significance of nuclear YY1 expression in breast cancer (BC), patients were stratified into high- and low-YY1-expression groups according to the cutoff value. Kaplan–Meier survival analyses were then performed to estimate overall survival (OS), and the differences between groups were statistically measured by log-rank tests. Our results showed clearly resolved survival curves, suggesting distinct clinical outcomes depending on YY1 expression levels. In Figure 3, representative immunohistochemical (IHC) images are provided to illustrate the range of YY1 nuclear staining patterns observed in tumor samples. As shown in Figure 3a, tumors categorized within the low-YY1-expression group displayed only weak and scattered nuclear staining, often limited to a small fraction of malignant cells. In contrast, tumors assigned to the high-YY1-expression group (Figure 3b) exhibited strong, diffuse nuclear staining across the majority of tumor cells, reflecting marked YY1 upregulation in cancer cell nuclei. These representative images highlight qualitative differences in nuclear YY1 expression, forming the basis for survival analyses.

**Figure 4:**
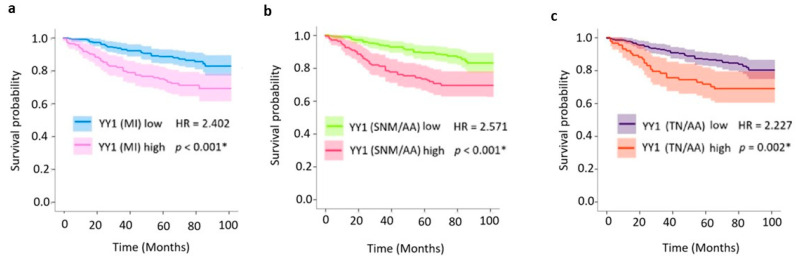
Patient stratification according to overall survival. Kaplan–Meier curves of the three most significant variables show YY1 expression by DP analysis: (**a**) medium intensity (MI), (**b**) strong nuclei plus mean/average area (SNM/AA), and (**c**) total nuclei per average area (TN/AA). * Statistically significant.

Patients with high nuclear YY1 expression showed a significantly reduced OS. The median OS was not reached; however, Kaplan–Meier plots showed on average an almost 20% decrease in OS at five years for the high-YY1 subset at all three selected variables with hazard ratios (HRs) above 2 (*p* < 0.001 and *p* = 0.002, Figure 4). For example, considering the medium intensity (MI) score, high-YY1 vs. low-YY1 patients exhibited the following survival rates at different time points: 92 vs. 99% at 12 months (1 yr), 80 vs. 93% at 36 months (3 years), and 74 vs. 88% at 60 months (5 years) (Figure 4a).

Univariate OS analysis in relation to clinical and pathological variables is summarized in Table 3. Several factors were significantly associated with OS, including molecular subtype, positive hormone receptor status (ER+ and PR+), nuclear grade, tumor size, axillary lymph node dissection, recurrence, prognostic stage (II–IV), and YY1 expression. Given that YY1 was significantly enriched in TNBC, we tested its prognostic value in this subset. However, probably due to the small number of patients within this subgroup, our results were not statistically significant (HR = 1.317, *p* = 0.566).

To determine independent predictors of survival, we performed multivariate Cox regression analysis (Table 4). Variables statistically significant after this step were molecular subtype, ER+ but not PR+ status, prognostic stage III, recurrence, ALND, and, interestingly, nuclear YY1 expression.

**Figure 5:**
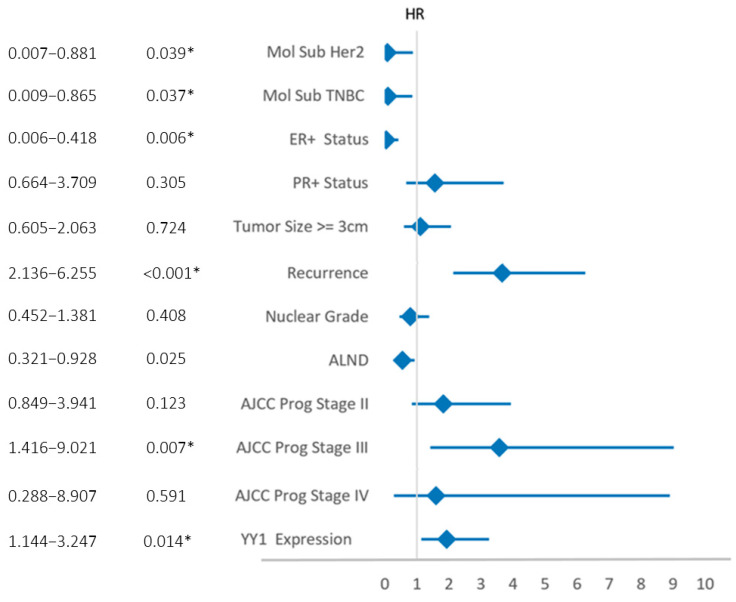
Forest plot of multivariate Cox regression analysis of OS of BC patients in our cohort. Only variables with 95% CIs not crossing the vertical axis are statistically significant. Hazard ratios and *p*-values are included for each variable. * Statistically significant.

YY1 was confirmed as an independent prognostic factor, with a hazard ratio (HR) of nearly two, indicating that high-YY1 patients have an almost 200% higher risk of death than the low-YY1 subset. The multivariate results are summarized graphically as a forest plot in Figure 5. These findings suggest that high nuclear YY1 expression in tumor areas measured by DP may serve as a reliable prognostic biomarker in BC.

**Figure 6:**
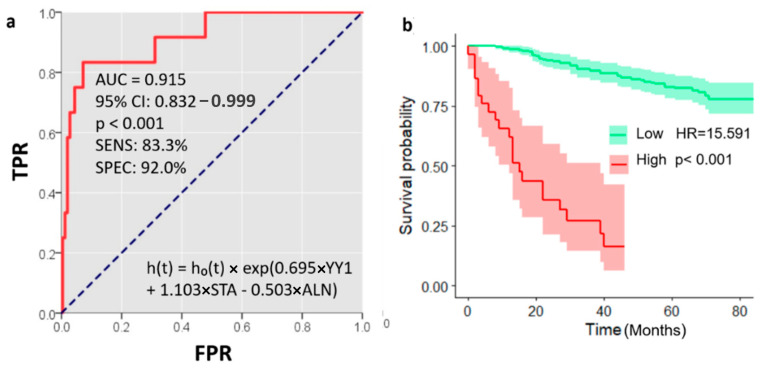
Mathematical modeling of multiple predictors: (**a**) ROC curve displaying enhanced prognostic value of combined scores constructed from significant variables in multivariate Cox regression. TPR: True-Positive Rate, FPR: False-Positive Rate, AUC: area under the curve, 95% CI: confidence interval, SENS: sensitivity, SPEC: specificity, h(t): hazard function at time *t*, h_0_(t): baseline hazard function, exp: exponential term, YY1: protein expression (medium intensity), STA: AJCC prognostic stage, ALN: short acronym for axillary lymph node dissection (ALND). (**b**) Overall survival analysis by Kaplan–Meier curve based on the mathematical model (combined score), including 95% CIs (confidence intervals) (shaded bands); HR: hazard ratio.

Additionally, we developed a mathematical model based on the multivariate Cox regression analysis, including two significant variables other than YY1, i.e., AJCC prognostic stage (STA) and axillary lymph node dissection (ALN), with the following equation: h(t) = h_0_(t) × exp (0.695 × YY1 + 1.103 × STA − 0.503 × ALN). ROC analysis of this model showed a better AUC of 0.915, similar sensitivity (83.3%), and much higher specificity (92%) (Figure 6a). This model also reflected better patient stratification in the Kaplan–Meier curve built for survival analysis (Figure 6b). Patients with a high score had a shorter overall survival at 1 year, 62 vs. 99%; 3 years, 27 vs. 90%; and 5 years, 0 vs. 83%.

### 2.4. Pan-Cancer and Genomic Analyses of YY1 Expression and Prognostic Impact on Breast Cancer

To better understand the broader relevance of YY1 in oncogenesis, we conducted a comprehensive pan-cancer analysis to evaluate gene expression across multiple cancer types. This analysis was performed using the TNM plot tool, which integrates RNA-seq datasets from major public repositories, including Gene Expression Omnibus (GEO), Genotype-Tissue Expression (GTEx), The Cancer Genome Atlas (TCGA), and Therapeutically Applicable Research to Generate Effective Treatments (TARGET).

Our findings revealed a significant overexpression of YY1 in breast cancer and many other malignancies, including acute myeloid leukemia (AML), colon cancer, esophageal cancer, liver cancer, lung adenocarcinoma and squamous cell carcinoma, ovarian cancer, bladder cancer, cervical cancer, and head and neck cancer (Supplementary Figure S3). Notably, YY1 was downregulated only in thyroid carcinoma, suggesting a potential tissue-specific regulation or tumor suppressor role in this context. This widespread overexpression across diverse cancer types supports the hypothesis of YY1 playing fundamental roles in tumor biology, particularly in promoting proliferation and survival pathways.

**Figure 7:**
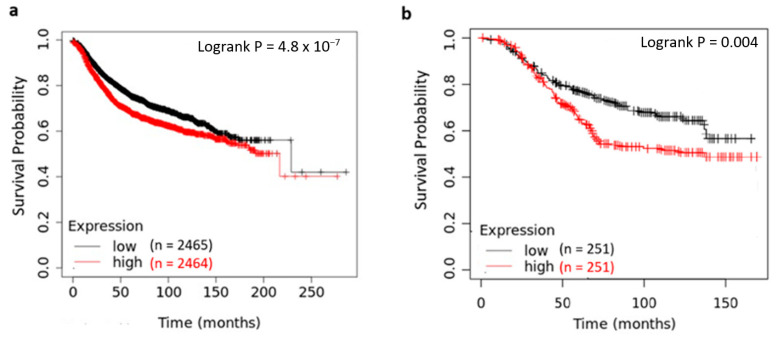
Survival analysis by Kaplan–Meier curves and log-rank tests based on YY1 expression in gene chip data from public web tools. (**a**) KM plotter. (**b**) GENT2.

In addition to gene expression profiles, we explored the prognostic impact of YY1 in breast cancer using the KM plotter and GENT2 platforms. These tools include survival data from cDNA microarray technology. Survival analyses (Figure 7) showed that high YY1 levels were associated with a significantly worse OS in breast cancer patients. Figure 7a shows mRNA expression stratified by YY1 level in a large patient cohort (*n* = 4929) and a follow-up over 21 years. Patients with high vs. low YY1 expression showed a median survival of 188 (15.6) vs. 230 months (19.2 years). Figure 7b shows another comparison from a different database with 502 patients, displaying differences in median survival of 120 months vs. median not reached for high- and low-YY1 subsets, respectively, whereas at 5-years, 63 vs. 77% of patients were alive, respectively.

**Figure 8:**
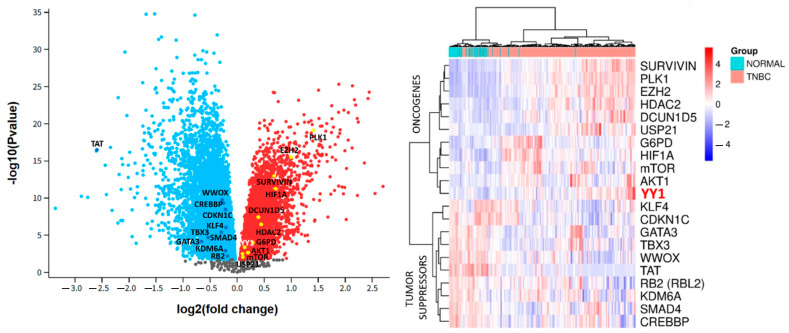
Volcano plot and heatmap of differentially expressed oncogenes and tumor suppressors directly or potentially interacting with YY1 in tumor growth and development. Clinical biopsies included TNBC vs. normal matched tissue. Expression scale presented in red and blue depicts up- and downregulated genes. Transcriptomic data (GSE21257) were obtained from comprehensive analysis of TNBC by gene expression microarrays from the GEO database. Red font highlights YY1 as our target gene.

We also found multiple oncogenes related to YY1 whose mRNA expression was upregulated (Figure 8) in TNBC vs. normal tissue, including Survivin (BIRC5), polo-like kinase 1 (PLK1), Enhancer of Zeste Homolog 2 (EZH2), Histone Deacetylase 2 (HDAC2), defective in cullin neddylation 1 domain containing 5 (DCUN1D5), ubiquitin specific peptidase 21 (USP21), glucose-6-phosphate dehydrogenase (G6PD), alpha subunit of hypoxia-inducible factor 1 (HIF1A), mammalian target of rapamycin (mTOR), and RAC-alpha serine/threonine-protein kinase (AKT1). Likewise, different tumor suppressor genes interacting with YY1 were downregulated in TNBC vs. normal tissue, such as Kruppel-like factor 4 (KLF4), cyclin dependent kinase inhibitor 1C (CDKN1C), GATA binding protein 3 (GATA3), T-box transcription factor 3 (TBX3), WW domain-containing oxidoreductase (WWOX), Tyrosine Aminotransferase (TAT), retinoblastoma-like protein 2 (RB2), lysine-specific demethylase 6A (KDM6A), SMAD Family Member 4 (SMAD4), and CREB-binding protein (CREBBP).

As can be seen in Figure 8, heatmap and volcano plot analyses showed a relatively modest increase in gene expression for YY1 and several oncogenes, such as USP21, mTOR, AKT1 G6PD, and HDAC2, and the same for some negatively regulated tumor suppressors, including RB2, KDM6A, GATA3, SMAD4, and TBX3. This was a general pattern, with the exception of the TAT gene showing a relatively larger fold change below −2.5 in absolute value. However, small, coordinated changes in different proteins directly interacting with YY1 could underlie its differential role in BC development.

Together, these data support a model in which YY1 may contribute to BC progression given its overexpression in tumor vs. normal tissue, association with more aggressive tumor and clinical phenotypes, and poor survival outcomes, making it a promising molecular biomarker and potential target for future diagnostic and therapeutic strategies.

## 3. Discussion

Breast cancer (BC) remains the second most frequently diagnosed malignancy worldwide and the, fourth leading cause of cancer-related death among women, according to the Global Cancer Observatory (GCO, 2024) [https://gco.iarc.fr/today, accessed on 8 September 2025]. Although mortality rates have declined since 1989 due to early detection and improved therapies [35], a subset of patients still develops resistance to chemotherapy [36]. Moreover, patients sharing the same clinical diagnosis and treatment regimen can exhibit markedly different outcomes, especially in underrepresented populations, due to tumor molecular heterogeneity [37]. This underscores the urgent need for new diagnostic and prognostic strategies.

The transcription factor YY1 has gained attention as a potential oncogenic driver, given its upregulation in several tumor types, including acute myeloid leukemia [12] and colon [15], liver [13], and breast cancers [19]. Here, we confirmed YY1 overexpression in tumor vs. normal tissue, consistent with previous reports in BC [22]. A particularly interesting finding in our study was strong nuclear YY1 expression in tumor-infiltrating immune cells. This observation aligns with that in functional studies of melanoma, where YY1 was found to positively regulate immune checkpoint receptors, such as PD-1, LAG3, and TIM-3, while suppressing type I cytokines IL-2 and IFN-γ, promoting T-cell exhaustion and immune evasion [38].

YY1 expression was found across all BC molecular subtypes in our cohort, but YY1-positive tumor cells were enriched in TNBC. This supports prior reports indicating that YY1 promotes proliferation, drug resistance, and epithelial–mesenchymal transition (EMT) in TNBC cell lines through various mechanisms, including the repression of pro-apoptotic genes like JAW [25] and the upregulation of lncRNAs such as DUXAP8 [26]. YY1 also induces EMT via SETD7, a process associated with metastasis and poor prognosis [23].

However, the prognostic role of YY1 in breast cancer remains controversial. In luminal tumors, YY1 acts as a transcriptional coactivator of ERα, enhancing tumor growth, correlated with poor prognosis [39]. Yet another study reported that nuclear YY1 expression was associated with favorable prognosis in luminal ER-positive tumors, possibly due to co-expression with AP-2α/β and their synergistic role with the estrogen receptor [40].

In TNBC, the literature is also controversial. While many studies suggest that YY1 acts as an oncogene, some evidence points to a protective role. For example, YY1 overexpression was associated with the suppression of LINC00152, a long non-coding RNA whose high levels correlate with ER/PR negativity, lymphatic invasion, advanced TNM stage, and reduced OS [41]. Therefore, YY1 may play dual roles depending on the molecular context and interacting partners.

Despite the discrepancies reported in the literature, our findings provide strong evidence for nuclear YY1 expression as an independent prognostic factor for poor OS in breast cancer patients. In our Mexican cohort, multivariate Cox regression analysis revealed that high nuclear YY1 levels were significantly associated with an almost 2-fold increased risk of death (HR = 1.927, 95% CI: 1.144–3.247, *p* = 0.014). Notably, this association remained significant even after adjusting for traditional clinicopathological parameters, such as tumor size, lymph node involvement, and molecular subtype, highlighting the robust prognostic value of YY1 beyond standard clinical variables.

Furthermore, we constructed a mathematical model derived from some independent factors in multivariate Cox regression, improving the accuracy of our predictions. These variables, besides YY1, were AJCC prognostic stage (STA) and axillary lymph node dissection (ALN), with the following equation: h(t) = h_0_(t) × exp (0.695 × YY1 + 1.103 × STA − 0.503 × ALN). The ROC curve for this model displayed a higher AUC of 0.915, similar sensitivity (83.3%), and enhanced specificity (92%). This model showed better discrimination of patient subgroups in the Kaplan–Meier curve for OS. AJCC prognostic stage is a key prognostic factor in BC, showing a highly accurate outcome prediction often combined with other biological variables, such as progesterone (PR) and/or estrogen receptor (ER) status, as well as HER2 status and tumor grade [42]. ALND is a diagnostic and therapeutic intervention used in breast cancer to evaluate the extent of cancer dissemination, contributing to cancer staging and guiding clinical decisions such as chemo- or radiotherapy plus targeted, immunological, and hormonal treatments. ALND implies the surgical removal of lymph nodes, thereby reducing the risk of recurrence [43], although it is gradually replaced by sentinel lymph node biopsy [44]. Combinations of these prognostic and therapeutic variables with novel molecular factors such as YY1 may enhance risk stratification and future therapeutic approaches rather than individual predictors.

Additionally, transcriptomic data from public databases indicate that several oncogenes and tumor suppressors related to YY1 in multiple processes associated with tumor growth and development are up- and downregulated in BC vs. normal samples. This could imply relevant biological implications in BC pathogenesis. Despite a pattern of relatively modest fold changes in the expression of multiple oncogenes and tumor suppressors interacting directly with YY1, their synergistic action can result in a more aggressive BC phenotype, as well as a reduced OS.

For instance, USP21 oncogenic activity mediates YY1 deubiquitination, preventing degradation, thereby increasing stability. This interaction promotes cancer cell proliferation, migration, and invasion [45]. YY1 can also stimulate proliferation by direct interaction with AKT, activating mTORC2-mediated AKT phosphorylation [46]. At the metabolic level, YY1 enhances cell growth via the transcriptional activation of G6PD, a key enzyme in the pentose phosphate pathway [47]. However, YY1 overexpression can inhibit tumor suppressor activity of the retinoblastoma protein (Rb), stimulating cell cycle progression [48]. The dysregulation of histone lysine demethylases (KDMs) can lead to tumorigenesis. For example, YY1 can recruit KDM6A to the promoter of neurotrophic receptor tyrosine kinase 1 (NTRK1), driving its upregulation and imatinib resistance [49]. Furthermore, YY1 can directly interact with members of the Smad family, such as Smad4, through the conserved N-terminal Mad homology 1 domain, causing the dose-dependent repression of the TGF-β pathway and its antiproliferative response [50].

Additional genes directly interacting with YY1 include HDAC2, a known oncogene in multiple malignancies. For example, a recent study showed that HDAC2, YY1, and c-Myc participate in lung cancer growth and development. HDAC2 and YY1 expression was elevated in cell lines and lung tumor samples, whereas HDAC2 inhibition decreased YY1 levels, and both played a role in cell migration and proliferation [51]. Another publication demonstrated that YY1 activates DCUN1D5 acting on the PI3K/AKT pathway, which stimulates cancer progression in TNBC patients. This novel transcriptional target of YY1 was identified as a potential biomarker and therapeutic target given its role in BC cell proliferation and invasion [31]. YY1 also stimulates the FASN-HIF1α pathway, causing ferroptosis suppression and the growth of ovarian cancer by inducing USP43 [52]. Many of these interactions and novel YY1 targets have not been explored in BC, which may help elucidate how the YY1 interactome could trigger its differential responses.

A major strength of our study lies in the integration of DP with TMAs, which enabled precise and reproducible quantification of nuclear YY1 protein expression. This approach allowed us to selectively analyze only tumor epithelial cells, excluding surrounding stromal or immune cells when necessary. As opposed to whole-section analysis or non-digitized IHC scoring, this methodology minimizes interobserver variability and sampling bias, enhancing the accuracy of biomarker evaluation. This level of spatial resolution is critical given that YY1 is also expressed in non-tumor cells within the tumor microenvironment, including infiltrating lymphocytes and fibroblasts. Traditional studies, such as those by Yao et al. [22], although foundational, often relied on semi-quantitative manual scoring across larger sections, which may have diluted tumor-specific YY1 signals with non-specific background expression. This could partially explain the inconsistencies in prior reports regarding YY1’s prognostic value. Moreover, by utilizing DP software, we achieved a standardized scoring system across hundreds of TMA cores, enabling us to stratify patients based on quantitative thresholds of nuclear YY1 intensity and distribution. This methodological rigor reinforces our conclusion that nuclear-localized YY1, and not total or cytoplasmic expression, is the most relevant variable in predicting poor clinical outcomes.

This tumor-specific, digital quantification approach is especially relevant for underrepresented populations, such as Latin American patients, with scarce molecular data and high heterogeneity. By demonstrating how YY1 maintains prognostic value in a distinct population, we contribute valuable insights validating YY1 as a biomarker and emphasize the relevance of incorporating advanced pathology tools such as TMAs and DP into future translational cancer research. Furthermore, pan-cancer analyses using TNMplot and survival in the KM plotter and GENT2 tools confirmed YY1 overexpression across multiple cancer types—including colon cancer, esophagus cancer, AML, ovarian cancer, lung adenocarcinoma, and BC—whereas high YY1 expression at transcriptomic level correlates with poor survival in BC patients. For the first time, our findings highlight YY1 as an independent prognostic factor, indicating that high nuclear YY1 expression in tumor areas measured by DP may serve as a reliable prognostic biomarker in BC.

Nevertheless, some limitations should be acknowledged. The study cohort consisted entirely of Mexican patients, which may reduce the generalizability of the findings to other populations. In addition, although focusing on tumor regions highlights the biology of cancer cells, it does not capture potential contributions from stromal or immune compartments within the tumor microenvironment. Finally, as a retrospective single-cohort study, these results would benefit from validation in larger, independent, and prospective datasets.

## 4. Materials and Methods

### 4.1. Subjects

The Ethical and Research Committees of the “Hospital General Regional No. 1”, “Instituto Mexicano del Seguro Social de Morelia”, and “Hospital General Dr. Miguel Silva” (Morelia, Michocán México) approved the protocol for this study (R-2020-1602-014). Breast cancer biopsies were obtained from 276 women, 13 of them with matched normal tissue obtained at institutions between 2015 and 2017. We gathered clinicopathological data from medical records, such as age, histological and nuclear grades, tumor size, axillary lymph node dissection (ALND), hormone receptor (i.e., estrogen (ER) and progesterone (PR)) status, and epidermal growth factor receptor 2 (HER2) status. Molecular subtypes (including luminal BC, HER2-enriched BC, and TNBC), AJCC (American Joint Committee en Cancer) prognostic stage, and disease recurrence were also included. OS data were measured from the initial diagnosis to the date of death or last follow-up. This cohort was previously used by our group to evaluate another biomarker [34].

### 4.2. Tissue Microarray

Representative areas of each specimen were identified by an expert pathologist, and a TMA was created using a Chemicon Advanced Tissue Arrayer (ATA 100, Chemicon Temecula, CA, USA). As previously described [53,54], the TMA was constructed from a donor sample paraffin-embedded into a recipient tissue block. The donor samples consisted of 3 mm high cylinders with a 0.4 mm needle to generate a 12 × 6 matrix. The TMA included three cores (spots) for each patient. After each block was completed, 4 μm thin sections were cut with a rotating microtome.

### 4.3. Immunohistochemical Analysis

For YY1 expression analysis, IHC staining was performed on the TMA sections. Briefly, the sections were deparaffinized and rehydrated in a gradient of decreasing ethanol concentrations. To unmask epitopes, antigen retrieval was performed with 10 mM sodium citrate solution (pH 6) by heating the sections under pressure. Endogenous peroxidases were blocked with 10% hydrogen peroxide, and nonspecific binding was blocked by incubating the sections in 2% porcine serum for 60 min at room temperature in a humid chamber. The sections were incubated with primary antibody vs. YY1 (Cat NBP2-20932, Novus Biologicals, Centennial CO, USA) overnight at 4 °C. Subsequently, the sections were incubated with secondary antibody ImmPRESS HRP Horse Anti-Rabbit IgG Polymer Detection Kit Peroxidase from Vector Laboratories (Burlingame, CA, USA) for 30 min. Reaction products were visualized with 3,3′-diaminobenzidine-H_2_O_2_ substrates (Vector Laboratories). The sections were counterstained with hematoxylin and dehydrated and mounted with Entellan resin.

### 4.4. Digital Pathology Analysis and Automated Image Quantification

DP-assisted analysis was performed by digitizing the TMAs, as previously described [53]. YY1-treated slides were scanned using an Aperio ScanScope CS2 slide scanner (Leica Biosystems, Buffalo Grove, IL, USA) at 20× magnification. Regions of interest were selected by an expert pathologist and manually annotated using Aperio ImageScope software (version 6.25 Leica Biosystems, Deer Park, IL, USA). YY1 staining was analyzed using Aperio ImageScope software with the Aperio nuclear algorithm (version 9.2, Leica Biosystems), without modifications. Default settings for the nuclear algorithm were used (“Nuclear Algorithm, User’s Guide” Leica Biosystems, MAN-0338, Revision 8; 5 August 2015). The algorithm measured stain intensity (brown signal) for the entire section, and a color labeling image was obtained for each TMA based on the nuclear staining intensity. Inputs were pre-configured for color quantification with the following thresholds: [minimum nuclear size (um^2^) = 10; maximum nuclear size (um^2^) = 1000; minimum roundness = 0.01; minimum compactness = 0; minimum elongation = 0.4; weak threshold (1+) = 220; moderate (2+) threshold = 210; strong threshold (3+) = 195]. Color saturation and its intensity in positive pixels for YY1 was classified by software algorithms as negative (blue), weak (yellow), moderate (orange), and strong (red). The results were scored as staining intensities from 0 to 3, and statistical analyses were performed using mean values. Data are presented as nuclear intensity/µm^2^.

### 4.5. Public Datasets

YY1 expression and prognostic value were analyzed using web-based tools that gather information from genomic databases such as TCGA, GEO, and EGA. To this end, we explored gene chip data from the Kaplan–Meier plotter [55] (last accession: 1 August 2023) and GENT2 expression database [56] (last accession: 1 August 2023). To evaluate the impact on the OS of breast cancer patients, YY1 expression was analyzed in 4929 subjects from KMplotter and 502 cases from GENT2. An additional pan-cancer analysis of YY1 transcript levels was performed on RNAseq data of matched tumor/normal biopsies through the TNMplot web tool [57] (last accession: 1 August 2023). We also constructed a volcano plot and heatmap based on the expression profiles of different oncogenes and tumor suppressors directly or potentially interacting with YY1. These genes related to cellular processes, such as cell proliferation, cell cycle, apoptosis, metabolism, and histone modification, were contrasted in TNBC vs. matched normal samples. Trancriptomic data were retrieved from the Gene Expression Omnibus (GEO) database (GSE76250) [55] (last accession: 1 August 2023) and processed in R language version 4.4.1 (Vienna, Austria).

### 4.6. Data Availability Statement

Gene expression and survival information obtained from public datasets and evaluated in the present study can be accessed through multiple genomic databases: KM plotter (http://kmplot.com/), GENT2 (http://gent2.appex.kr/gent2/), and TNMplot (https://tnmplot.com/analysis/) (all were last accessed on 1 August 2023).

### 4.7. Statistical Analysis

YY1’s value as a prognostic biomarker in breast cancer was evaluated by constructing receiver operator characteristic (ROC) curves. Through statistical significance and area under the curve (AUC), we selected relevant variables from the DP data. Patients were stratified into low and high YY1 levels based on the optimal cutoff. Then, categorical associations with clinicopathological variables were evaluated by Chi-Squared and Fisher’s Exact tests. YY1’s relationships with clinical features, as continuous variables, were calculated by Mann–Whitney U and Student’s *t*-tests. Mean ± standard deviation or median plus range were used to present the central tendency and dispersion depending on the data distribution. Normality testing of quantitative variables was performed by the Kolmogorov–Smirnov method (n ≥ 50). OS analysis at a uni/multidimensional level was performed by Kaplan–Meier curves using log-rank tests and Cox regression. All analyses were carried out in R programming language version 4.4.1 and SPSS software version 25.0 (IBM, New York, NY, USA), and *p* < 0.05 was considered statistically significant.

## 5. Conclusions

In summary, our findings highlight for the first time nuclear YY1 expression as a promising independent prognostic biomarker in BC, particularly in underrepresented populations, such as Latin American patients, when quantified using DP and a tumor-focused TMA analysis. However, due to the conflicting roles of YY1 in different breast cancer subtypes and cellular contexts, further research is needed to define its mechanistic contributions to tumor progression, immune modulation, and therapy resistance. DP platforms and integrative molecular profiling will be critical for resolving this controversy and unlocking YY1 as a potential clinically actionable target. The development of a mathematical model combining YY1 as a molecular predictor with other prognostic factors in a comprehensive panel could significantly improve risk stratification and guide further clinical decisions in breast cancer.

## Figures and Tables

**Table 1:** Clinicopathological characteristics of 276 patients with breast cancer.

Characteristics	n (%)	Total (n = 276)	*p*-Value
Age	276		<0.001 *
<50 yr		111 (40.2%)	
≥50 yr		165 (59.8%)	
Histologic Grade	273		<0.001 *
I		59 (21.6%)	
II		146 (53.5%)	
III		68 (24.9%)	
Nuclear Grade	273		<0.001 *
1		25 (9.2%)	
2		171 (62.6%)	
3		77 (28.2%)	
Border Type	276		<0.001 *
Non-Infiltrative		206 (74.6%)	
Infiltrative		70 (25.4%)	
Tumor size	276		0.952
≤3 cm		139 (50.4%)	
>3 cm		137 (49.6%)	
ALND	276		<0.001 *
No		99 (35.9%)	
Yes		177 (64.1%)	
ER Status	276		<0.001 *
Negative		86 (31.2%)	
Positive		190 (68.8%)	
PR Status	276		0.003 *
Negative		113 (40.9%)	
Positive		163 (59.1%)	
HER2 Status	273		<0.001 *
Negative		227 (83.2%)	
Positive		46 (16.8%)	
Molecular Subtype	268		<0.001 *
LUM A + B		190 (70.9%)	
HER2		26 (9.7%)	
TNBC		52 (19.4%)	
AJCC Prognostic Stage	272		<0.001 *
I		103 (37.9%)	
II		101 (37.1%)	
III		62 (22.8%)	
IV		6 (2.2%)	
Recurrence	276		<0.001 *
No		215 (77.9%)	
Yes		61 (22.1%)	

All comparisons: Chi Square Test, ALND: Axillary Lymph Node Dissection, ER: Estrogen Receptor, PR: Progesterone Receptor, HER2: Human Epidermal Growth Factor Receptor 2, LUM: Luminal, TNBC: Triple-Negative Breast Cancer, AJCC: American Joint Committee on Cancer * Statistically significant (*p* < 0.05).

**Table 2:** Association between clinicopathological characteristics and YY1 protein expression.

Variable n (%)	N	Low YY1 (n = 165)	High YY1 (n = 111)	Total (n = 276)	*p*-Value
Age	276				1.000 ^C^
<50 yr		66 (40.0%)	45 (40.5%)	111 (40.2%)	
≥50 yr		99 (60.0%)	66 (59.5%)	165 (59.8%)	
Histologic Grade	273				0.415 ^C^
I		37 (22.7%)	22 (20.0%)	59 (21.6%)	
II		90 (55.2%)	56 (50.9%)	146 (53.5%)	
III		36 (22.1%)	32 (29.1%)	68 (24.9%)	
Nuclear Grade	273				0.547 ^C^
1		15 (9.2%)	10 (9.1%)	25 (9.2%)	
2		106 (65.0%)	65 (59.1%)	171 (62.6%)	
3		42 (25.8%)	35 (31.8%)	77 (28.2%)	
Border Type	276				0.400 ^C^
Non-		120 (72.7%)	86 (77.5%)	206 (74.6%)	
Infiltrative		45 (27.3%)	25 (22.5%)	70 (25.4%)	
Tumor size	276				0.037 ^C^*
≤3 cm		92 (55.8%)	47 (42.3%)	139 (50.4%)	
>3 cm		73 (44.2%)	64 (57.7%)	137 (49.6%)	
ALND	276				0.074 ^C^
No		52 (31.5%)	47 (42.3%)	99 (35.9%)	
Yes		113 (68.5%)	64 (57.7%)	177 (64.1%)	
ER Status	276				0.185 ^C^
Negative		46 (27.9%)	40 (36.0%)	86 (31.2%)	
Positive		119 (72.1%)	71 (64.0%)	190 (68.8%)	
PR Status	276				0.035 ^C^*
Negative		59 (35.8%)	54 (48.6%)	113 (40.9%)	
Positive		106 (64.2%)	57 (51.4%)	163 (59.1%)	
HER2 Status	273				0.414 ^C^
Negative		132 (81.5%)	95 (85.6%)	227 (83.2%)	
Positive		30 (18.5%)	16 (14.4%)	46 (16.8%)	
Molecular Subtype	268				0.026 ^F^*
LUM A + B		119 (75.3%)	71 (64.5%)	190 (70.9%)	
HER2		17 (10.8%)	9 (8.2%)	26 (9.7%)	
TNBC		22 (13.9%)	30 (27.3%)	52 (19.4%)	
AJCC Prog Stage	272				0.003 ^F^*
I		74 (45.7%)	29 (26.4%)	103 (37.9%)	
II		58 (35.8%)	43 (39.1%)	101 (37.1%)	
III		27 (16.7%)	35 (31.8%)	62 (22.8%)	
IV		3 (1.9%)	3 (2.7%)	6 (2.2%)	
Recurrence	276				0.554 ^C^
No		131 (79.4%)	84 (75.7%)	215 (77.9%)	
Yes		34 (20.6%)	27 (24.3%)	61 (22.1%)	

^C^: Chi Square Test, ^F^: Fisher’s Exact Test, ALND: Axillary Lymph Node Dissection, ER: Estrogen Receptor, PR: Progesterone Receptor, HER2: Human Epidermal Growth Factor Receptor 2, LUM: Luminal, TNBC: Triple-Negative Breast Cancer, AJCC: American Joint Committee on Cancer, * Statistically significant.

**Table 3:** Univariate Cox regression analysis of overall survival in BRCA.

		Univariate Analysis		
Variable	Category	HR	95% CI	*p* Value
Age	<50	1		
	≥50	1.040	0.36–1.700	0.875
Molecular Subtype	LUMINAL A + B	1		
	HER2	2.317	1.155–4.647	0.018 *
	TNBC	2.018	1.153–3.531	0.014 *
ER Status	Negative	1		
	Positive	0.504	0.311–0.819	0.006 *
PR Status	Negative	1		
	Positive	0.529	0.327–0.855	0.009 *
HER2 Status	Negative	1		
	Positive	1.004	0.525–1.917	0.991
Tumor size	<3 cm	1		
	≥3 cm	1.852	1.132–3.028	0.014 *
Recurrence	No	1		
	Yes	3.963	2.445–6.424	<0.001 *
Nuclear Grade	1 + 2	1		
	3	1.748	1.060–2.882	0.029 *
ALND	No	1		
	Yes	0.459	0.284–0.742	0.001 *
AJCC Prognostic Stage	I	1		
	II	2.230	1.115–4.460	0.023 *
	III	5.882	2.973–11.637	<0.001 *
	IV	5.240	1.169–23.494	0.030 *
Histologic Grade	I + II	1		
	III	1.289	0.755–2.201	0.351
YY1 Protein Expression	Low	1		
	High	2.402	1.479–3.899	<0.001 *

ALND: Axillary Lymph Node Dissection, ER: Estrogen Receptor, PR: Progesterone Receptor, HER2: Human Epidermal Growth Factor Receptor 2, LUM: Luminal, TNBC: Triple−Negative Breast Cancer, AJCC: American Joint Committee on Cancer. * Statistically significant.

**Table 4:** Multivariate analysis of survival in breast cancer patients (Cox regression).

		Multivariate Analysis		
Variable	Category	HR	95% CI	*p* Value
Molecular Subtype	LUMINAL A + B	1		
	HER2	0.080	0.007–0.881	0.039 *
	TNBC	0.087	0.009–0.865	0.037 *
ER Status	Negative	1		
	Positive	0.050	0.006–0.418	0.006 *
PR Status	Negative	1		
	Positive	1.569	0.664–3.709	0.305
Tumor size	<3 cm	1		
	≥3 cm	1.117	0.605–2.063	0.724
Recurrence	No	1		
	Yes	3.655	2.136–6.255	<0.001 *
Nuclear Grade	1 + 2	1		
	3	0.790	0.452–1.381	0.408
ALND	No			
	Yes	0.545	0.321–0.928	0.025
AJCC Prognostic Stage	I	1		
	II	1.829	0.849–3.941	0.123
	III	3.575	1.416–9.021	0.007 *
	IV	1.602	0.288–8.907	0.591
YY1 Protein Expression	Low	1		
	High	1.927	1.144–3.247	0.014 *

ALND: Axillary Lymph Node Dissection, ER: Estrogen Receptor, PR: Progesterone Receptor, HER2: Human Epidermal Growth Factor Receptor 2, LUM: Luminal, TNBC: Triple−Negative Breast Cancer, AJCC: American Joint Committee on Cancer. * Statistically significant.

## Data Availability

The datasets analyzed for this study in three different hospitals can be obtained directly from the corresponding author.

## Supplementary Materials

The following supporting information can be downloaded at: https://www.mdpi.com/article/10.3390/ijms26188777/s1.
