# Supplementary material for: Clinical Significance of Nuclear Yin-Yang Overexpression Evaluated by Immunohistochemistry in Tissue Microarrays and Digital Pathology Analysis: A Useful Prognostic Tool for Breast Cancer
Source: Int J Mol Sci. 2025 Sep 9;26(18):8777. doi: 10.3390/ijms26188777 (PMC12469722; doi:10.3390/ijms26188777)
Supplement: Supplementary file 1 [file ijms-26-08777-s001.zip › ijms-3811994-supplementary.pdf]

Supplementary Figure 1

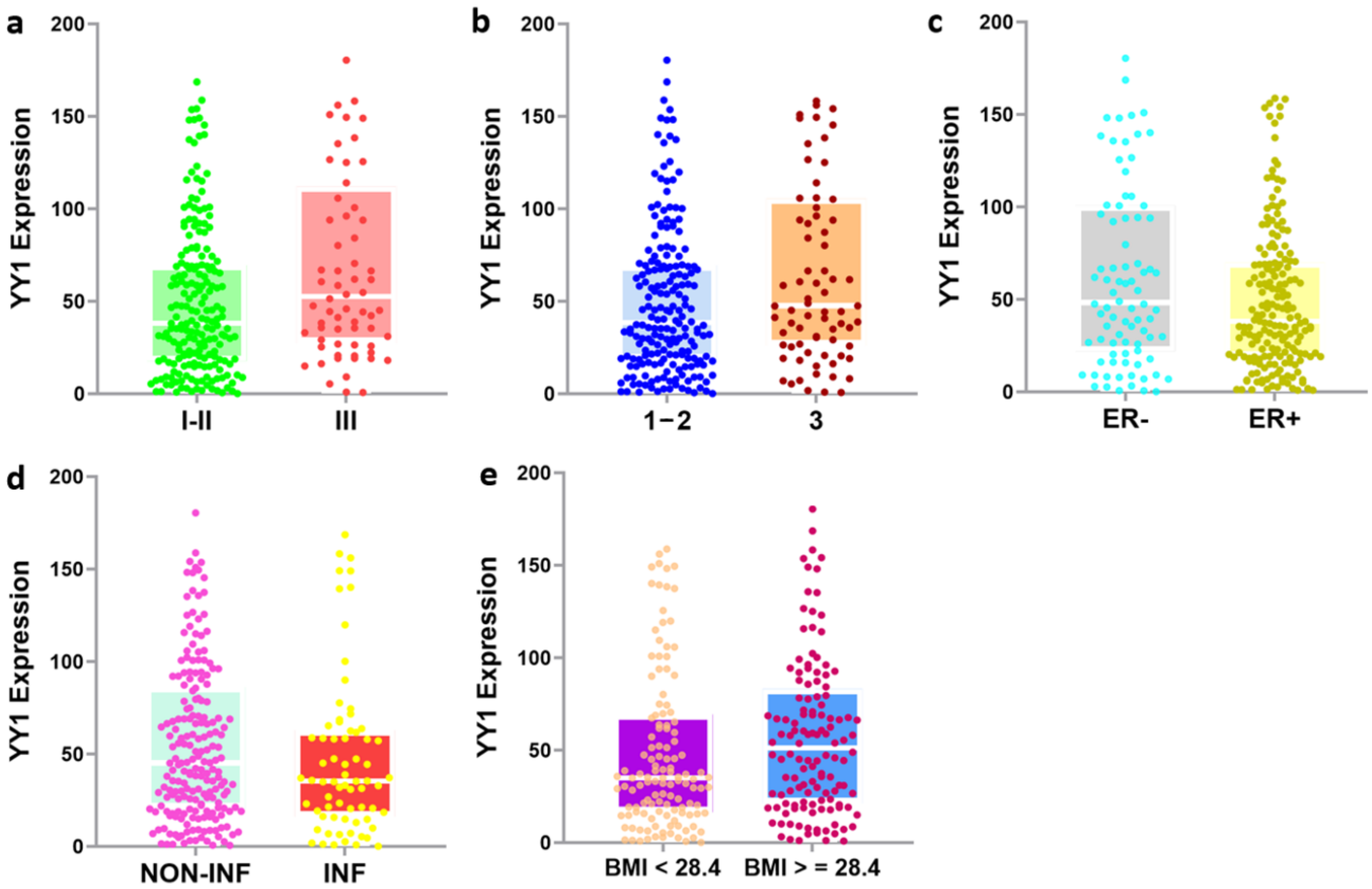

Relationship between clinicopathological characteristics and YY1 expression as continuous variable. a) Histological grade (p =0.005) b) Nuclear grades (p =0.022) c) Estrogen Receptor (p =0.097) d) Border Type (non infiltrative and infiltrative) (p =0.085) e) Body mass index (p =0.052).

Supplementary Figure 2

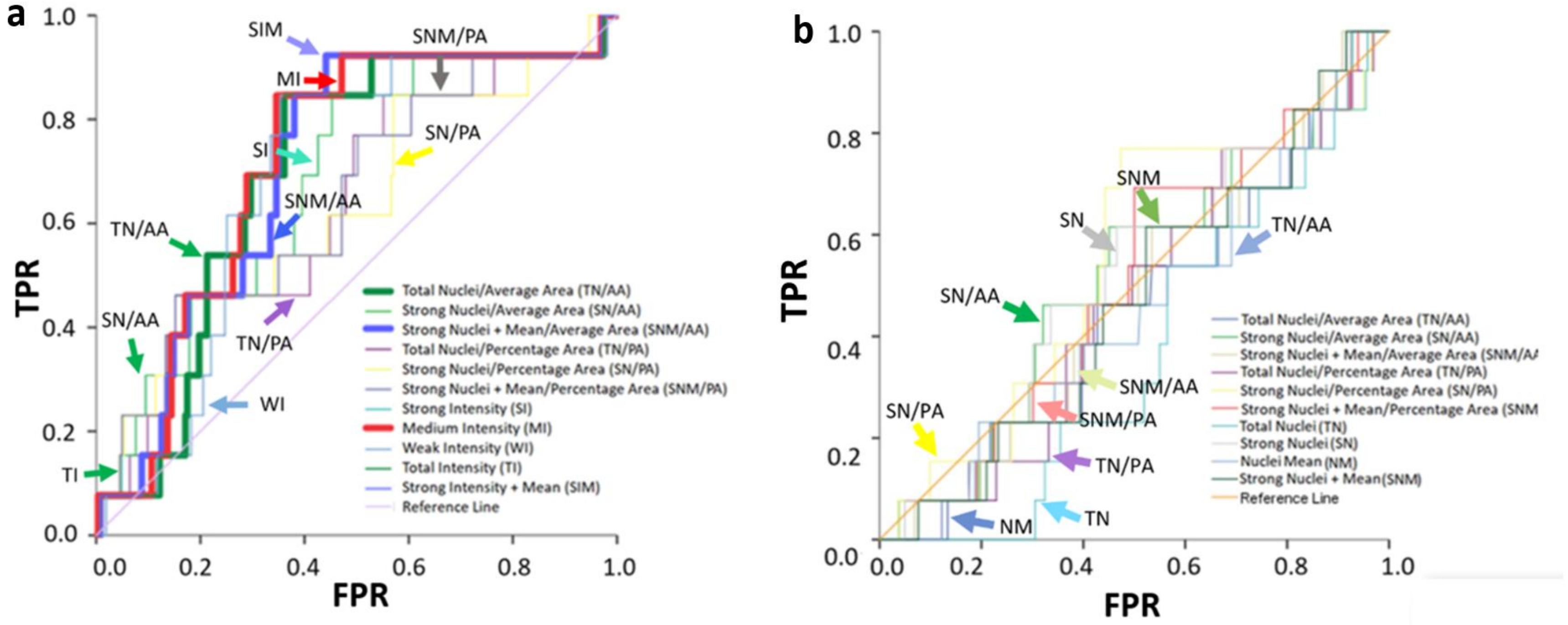

Multiple ROC curves for variables of YY1 quantification via DP. **A)** Tumor region: relevant ROC curves selected in terms of area under the curve (AUC) and p-value.. **B)** Whole spot: ROC curves of tumor + normal regions (not statistically significant). Different colored lines represent each variable measured by DP as indicated by arrows.

Supplementary Figure 3

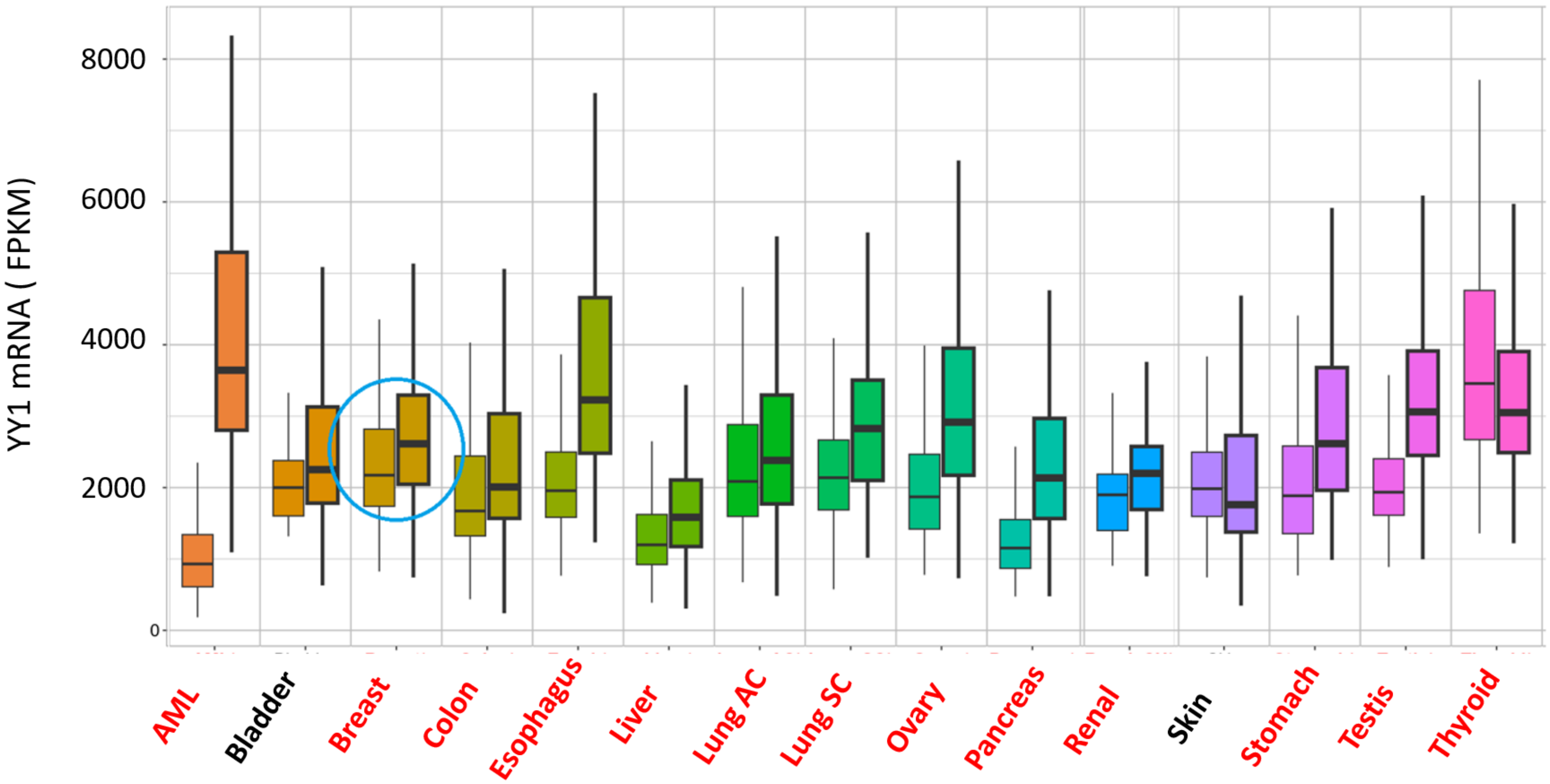

Pan-cancer analysis of YY1 mRNA expression across normal and tumor RNA Seq data from TNMplot (Database setup: GEO, GTex, TCGA, TARGET). Tissues with statistically significant differences are shown in red, whereas breast cancer vs. normal is circled in blue (Mann-Whitney U test). FPKM: Fragments Per Kilobase Million, AML: Acute Myeloid Leukemia, AC: Adenocarcinoma, SC: Squamous cell carcinoma.
